# Arthroscopic partial repair versus debridement combined with acromioplasty alone for irreparable rotator cuff tears in the elderly

**DOI:** 10.3389/fsurg.2025.1615015

**Published:** 2025-09-19

**Authors:** Ning Li, Lu Sun, Zhongyuan Zhang, Hanwei Kang, Hongjiang Jiang, Liwu Qin

**Affiliations:** Department of Minimally Invasive Joint, Shandong Wendeng Osteopathic Hospital, Weihai, China

**Keywords:** irreparable rotator cuff tear, arthroscopy, partial repair, debridement, elderly

## Abstract

**Objective:**

To compare postoperative outcomes of arthroscopic partial repair vs. debridement combined with acromioplasty in elderly patients with irreparable rotator cuff tears, focusing on pain relief, functional improvement, and quality-of-life enhancement.

**Methods:**

Between January 2019 and March 2022, 41 patients (partial repair group, *n* = 21; debridement group, *n* = 20) with irreparable rotator cuff tears were prospectively enrolled. Functional outcomes [Constant-Murley Score [CMS], University of California Los Angeles Score [UCLA]) were assessed preoperatively and 12 months postoperatively. Visual Analog Scale (VAS) scores were recorded at 2 and 6 weeks. Magnetic resonance imaging (MRI) and anteroposterior x-rays were used to measure global fatty degeneration index (GFDI) and acromiohumeral distance (AHD). Tendon healing was evaluated using the Sugaya classification system.

**Results:**

All surgeries were completed without complications. Follow-up averaged 14.1 months (range, 12–18 months). Postoperative CMS (partial repair: 43.57–70.86 vs. debridement: 42.55–58.95) and UCLA scores (partial repair: 8.67–21.43 vs. debridement: 8.30–18.40) improved significantly in both groups (*P* < 0.05), with greater enhancements in muscle strength and range of motion favoring partial repair. VAS scores were higher in the partial repair group at 2 weeks (3.1 ± 0.8 vs. 2.1 ± 0.7, *P* < 0.05) but equivalent at 6 weeks (*P* > 0.05). Postoperative GFDI increased in both groups (*P* < 0.05) without intergroup differences. AHD remained stable in the partial repair group (*P* > 0.05) but decreased in the debridement group (*P* < 0.05), with higher AHD persisting in the partial repair subgroup (*P* < 0.05). Subgroup analysis revealed no differences in outcomes between re-tear and non-re-tear patients. Preoperative AHD correlated positively with postoperative CMS and UCLA scores (*P* < 0.05), while Sugaya classification and preoperative GFDI showed no association with functional outcomes.

**Conclusion:**

Arthroscopic partial repair yielded superior functional outcomes compared to debridement combined with acromioplasty in elderly patients with irreparable rotator cuff tears, particularly enhancing shoulder strength and range of motion while preserving AHD. Early postoperative pain should be anticipated. Preoperative AHD emerged as a predictor of functional recovery.

## 1 Introduction

Rotator cuff tears are the primary source of shoulder pain in elderly patients. These injuries frequently become irreparable due to factors such as early asymptomatic, inflammatory response to irritation and degeneration of the tendon, which prevents anatomical repair (1). Although several treatment options exist for irreparable rotator cuff tears, a universally accepted optimal treatment protocol remains elusive. Consequently, individualized treatment strategies must be tailored to each patient's condition and requirements (2).Both arthroscopic partial repair and debridement combined with acromioplasty are effective treatments (3) that can adequately address the needs of most elderly patients by alleviating pain and improving shoulder joint function to a meaningful degree. These interventions offer several advantages, including minimal invasiveness, rapid recovery, low cost and a short learning curve, rendering them the preferred treatment options for both elderly patients and surgeons. However, postoperative retear rates of 50%–60% (4, 5) raise concerns about the efficacy of partial repairs in certain elderly populations. This study aims to determine whether partial repair offers more significant benefits over debridement combined with acromioplasty alone in terms of postoperative pain relief, functional improvement and quality-of-life enhancement for elderly patients with irreparable rotator cuff tears.

## 2 Materials and methods

### 2.1 General data

From January 2019 to March 2022, forty-one elderly patients with irreparable rotator cuff tears were studied. They were randomly assigned to partial repair group (*n* = 21) or debridement group (*n* = 20) using a computer-generated random sequence, with allocation concealment ensured by sealed, opaque envelopes to reduce selection bias (Table 1).

**Table 1:** Comparison of baseline data between the two groups.

Baseline data	Partial repair (*n* = 21)	Debridement (*n* = 20)	Statistic	*P*-value
Age (M, years)	68.14 ± 4.35	67.15 ± 4.21	*t* = 0.742	0.462
Gender (Male/Female, cases)	11/10	9/11		0.758
Disease duration (M, months)	4.6 ± 1.3	4.8 ± 1.7	*t* = 0.387	0.701
VAS (x ± s, score)	6.92 ± 1.38	6.58 ± 1.51	*t* = 0.588	0.562
Preoperative CMS (x ± s, score)	43.57 ± 793	42.55 ± 8.86	*t* = 0.389	0.699
Preoperative UCLA (x ± s, score)	8.67 ± 2.48	8.30 ± 2.00	*t* = 0.520	0.606

Criteria for irreparable rotator cuff tears include: (1) Despite adequate soft tissue release during the operation, anatomical repair of the torn tendon and the footprint area cannot be accomplished (6); (2) Involvement of two or more tendons or a tear exceeding 5 cm in size; (3) Significant tendon retraction to the periphery of the glenohumeral joint, complicating attempts to reposition the tendon; (4) Magnetic resonance imaging revealing fatty infiltration over grade 3 (7).

Inclusion criteria: (1) Age >60 years; (2) Presence of day or night pain and shoulder dysfunction; (3) Underwent shoulder arthroscopy.

Exclusion criteria: (1) Prior shoulder surgery on the affected side; (2) Severe shoulder arthritis (Hamada type IV) (8); (3) Shoulder instability; (4) Nerve injury in the affected upper limb; (5) Bilateral rotator cuff tear; (6) Follow-up duration <12 months; (7) High expectations for postoperative shoulder functional recovery.

### 2.2 Surgical methods

**Figure 1:**
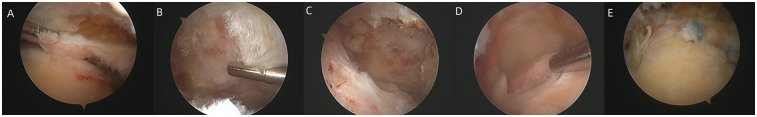
**(A)** Intraoperative exploration: A torn tendon failing to cover the footprint was considered irreparable. **(B)** Acromial osteophyte formation. **(C)** Post-acromioplasty view with preserved coracoacromial ligament. **(D)** Reverse contouring of the humeral greater tuberosity osteophyte. **(E)** Arthroscopic appearance after partial repair via anchor suturing.

Both groups underwent surgery performed by the same skilled surgeon and assistant. General anesthesia in combination with local nerve block anesthesia (brachial plexus within the interscalene groove formed by the anterior and middle scalene muscles) was administered to both groups. The surgeries for both groups were conducted with the patients positioned in lateral decubitus under traction. Various portals including posterior, anterior, and lateral portals to the shoulder were utilized during the procedures. Initially, using an endoscope, exploration was carried out to assess the rotator cuff tear and hyperosteogeny, leading to the determination that the torn tendons were irreparable (Figures 1A,B). In cases where the long head of biceps brachii tendon exhibited more than 1/4 injury, or dislocation and subluxation were present due to the extent of the injury, the tendon was excised. Conversely, if the injury was less than 1/4, debridement and trimming of the tendon were performed.

#### 2.2.1 Debridement group

In debridement group, thorough cleaning of the devitalized tendon tissue and surrounding synovial bursa was conducted, along with acromioplasty and greater tuberosity of the humerus procedures (Figures 1C,D). The integrity of the coracoacromial ligament was preserved.

#### 2.2.2 Partial repair group

In partial repair group, after debridement and acromioplasty, the adherent tendon tissue was released. Depending on the tear severity, side-to-side suturing and medial relocation of the insertion point towards the articular cartilage of the humeral head were performed to reduce the tear gap. A partial repair of the torn tendon was achieved by inserting an internal row anchor for suturing, oriented at a 90° angle to the bone surface. In cases of evident osteoporosis, the anchor could be directly screwed into the bone (Figure 1E).

### 2.3 Postoperative rehabilitation and follow-up

Patients in two groups underwent different rehabilitation protocols post-operation. The repair group received shoulder joint abduction brace fixation, oral calcium, and vitamin D. On the first day after surgery, they performed hand grip and elbow flexion and extension exercises on the affected limb. On the second day, they engaged in passive shoulder joint “pendulum” exercises. Between 6 and 8 weeks post-operation, they did passive shoulder joint exercises such as forward flexion, lifting, and external rotation. At 3 months post-operation, they performed active functional exercises using elastic bands on the affected limb. In contrast, patients in debridement group underwent shoulder pendulum exercises, passive activities, and active functional exercises post-operation.

Pain levels were assessed using the Visual Analogue Scale (VAS) at the 2-week and 6-week follow-up. The pain, function, and patient satisfaction were evaluated using the Constant-Murley Shoulder Function Scale (CMS) (9) and the University of California Los Angeles Scale (UCLA) (10) preoperatively and 12 months postoperatively.

**Figure 2:**
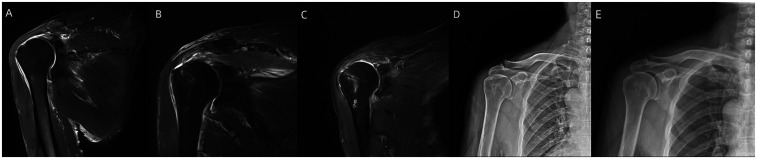
**(A)** Preoperative MRI findings of severe supraspinatus tendon tear with significant retraction indicate irreparability. **(B)** Postoperative MRI showed partial repair of the supraspinatus tendon with coverage of the humeral head. **(C)** MRI at the final follow-up showed good healing of the supraspinatus tendon. **(D,E)** Pre- and post-operative x-rays showed no significant reduction in the acromiohumeral distance (AHD) in partial repair group.

During the final follow-up, patients in partial repair group underwent magnetic resonance imaging (MRI) and anteroposterior x-ray. Tendon healing was evaluated using the Sugaya classification system (11). The steatosis grades of the supraspinatus, infraspinatus, and subscapularis muscles were measured pre- and post-operation to calculate the Global Fatty Degeneration Index (GFDI) (12). Additionally, the acromiohumeral distance (AHD) was measured (Figure 2).

Independent assessors not involved in intervention delivery or recruitment were blinded to group allocation and administered functional assessments throughout the study.

### 2.4 Statistical methods

Statistical analysis was conducted using SPSS 27.0. Univariate analysis utilized the Mann–Whitney *U*-test, *T*-test, and Fisher's exact test, depending on the data's normality. Preoperative and postoperative values were compared employing paired *T*-test or Mann–Whitney *U*-test. Pearson correlation analysis or Spearman's rank correlation analysis was employed to assess correlations. A *P*-value less than 0.05 indicates a statistically significant difference.

## 3 Result

41 patients underwent surgery without any infections or postoperative complications. Rehabilitation was conducted postoperatively, and the follow-up period ranged from 12 to 18 months (mean 14.1 months). All patients expressed satisfaction with the surgical outcomes during the final follow-up. Based on the Sugaya MRI healing grade, 14 patients in partial repair group experienced a retear (grade IV–V).

**Figure 3:**
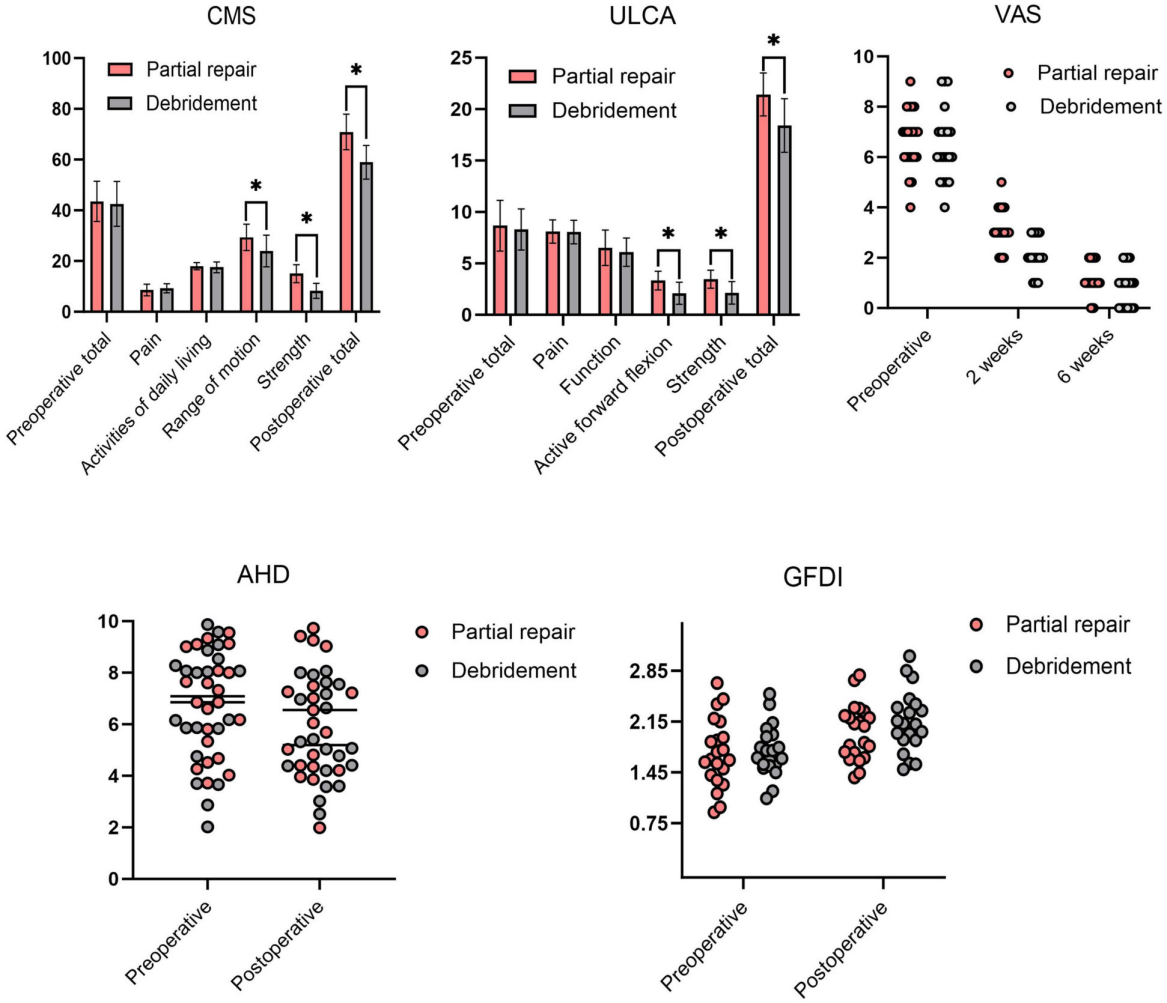
Comparison of outcomes and imaging data between the two groups. * means significant difference (*P* < 0.05).

The CMS (Partial repair group, 43.57–70.86; Debridement group, 42.55–58.95) and the UCLA scores (Partial repair group 8.67–21.43; Debridement group, 8.30–18.40) significantly improved in both groups. But the improvement was significantly greater in partial repair group than in debridement group especially in terms of muscle strength and range of motion (*P* < 0.05). Two weeks post-operation, the VAS scores were 3.1 ± 0.8 for partial repair group and 2.1 ± 0.7 for debridement group, showing a statistically significant difference (*P* < 0.05). At six weeks post-operation, the VAS scores were 1.1 ± .7 and 0.8 ± 0.8, respectively, with no significant difference (*P* > 0.05) (Table 2). GFDI significantly increased postoperatively compared to preoperative levels in both groups (*P* < 0.05), with no significant intergroup difference (*P* > 0.05). AHD remained unchanged before and after surgery in partial repair group (*P* > 0.05), while in debridement group, AHD significantly decreased postoperatively (*P* < 0.05). Additionally, AHD in partial repair group were significantly greater than that in debridement group (*P* < 0.05) (Table 3; Figure 3).

**Table 2:** Comparison of postoperative outcomes between the two groups.

Outcome measure	Category or time point	Partial repair	Debridement	Statistic	*P*-value
VAS	2 weeks	3.1 ± 0.8	2.1 ± 0.7	*t* = 4.407	*P* < 0.001
	6 weeks	1.1 ± 0.7	0.8 ± 0.8	*t* = 1.541	0.131
CMS	Total	70.86 ± 6.99	58.95 ± 6.62	*t* = 5.595	*P* < 0.001
	Pain	8.57 ± 2.32	9.25 ± 1.83	*t* = −1.038	0.306
	Activities of daily living	17.95 ± 1.43	17.50 ± 2.09	*t* = 0.812	0.422
	Range of motion	29.33 ± 5.19	23.95 ± 6.23	*t* = 3.013	0.005
	Strength	15.00 ± 3.54	8.25 ± 2.94	*t* = 6.633	*P* < 0.001
UCLA	Total	21.43 ± 2.09	18.40 ± 2.60	*t* = 4.119	*P* < 0.001
	Pain	8.10 ± 1.14	8.05 ± 1.15	*t* = 0.127	0.900
	Function	6.52 ± 1.72	6.10 ± 1.37	*t* = 0.869	0.390
	Active forward flexion	3.33 ± 0.91	2.10 ± 1.07	*t* = 3.975	*P* < 0.001
	Strength	3.48 ± 0.87	2.15 ± 1.09	*t* = 4.312	*P* < 0.001

**Table 3:** Comparison of imaging data between two groups.

Outcome measure	Partial repair	Debridement	Statistic	*P*-value
Preoperative GFDI	1.72 ± 0.47	1.75 ± 0.35	*t* = −0.230	0.819
Postoperative GFDI	2.00 ± 0.39^a^	2.15 ± 0.42^a^	*t* = −1.108	0.275
Preoperative AHD	6.84 ± 1.89	6.67 ± 2.33	*t* = 0.263	0.794
Postoperative AHD	6.55 ± 2.41^b^	5.57 ± 1.79^b^	*t* = 1.487	0.145

^a^ GFDI significantly increased postoperatively compared to preoperative levels in partial repair group (*P* = 0.037) and the debridement group (*P* = 0.002).

^b^ AHD before and after surgery did not show a significant difference in the partial repair group (*P* = 0.576), but did show a significant difference in the debridement group (*P* = 0.045).

In partial repair group, both non-re-tear and re-tear subgroups exhibited significant improvements in postoperative CMS and UCLA scores compared to preoperative values (*P* < 0.05). However, no statistically significant differences were observed in postoperative CMS or UCLA scores between the two subgroups (*P* > 0.05) (Table 4). Radiological assessment further revealed that, in the re-tear subgroup, postoperative GFDI were significantly higher than preoperative values (*P* < 0.05); conversely, no significant difference was found in preoperative vs. postoperative GFDI indices within the non-re-tear subgroup (*P* > 0.05). Additionally, there were no significant differences in GFDI between the two subgroups (*P* > 0.05). Similarly, neither subgroup showed a statistically significant change in AHD from pre- to post-operation (*P* > 0.05), and no inter-subgroup differences in AHD were observed (*P* > 0.05) (Table 5).

**Table 4:** Comparison of postoperative outcomes between re-tear group and non-re-tear group.

Outcome measure	Non-re-tear	Re-tear	Statistic	*P*-value
VAS	1.29 ± 0.49	1.07 ± 0.73	*t* = 0.698	0.494
CMS	73.71 ± 4.86	69.43 ± 7.59	*t* = 1.352	0.192
UCLA	21.57 ± 1.90	21.36 ± 2.24	*t* = 0.216	0.831

**Table 5:** Comparison of imaging data between re-tear group and non-re-tear group.

Outcome measure	Non-re-tear	Re-tear	Statistic	*P*-value
Preoperative GFDI	1.77 ± 0.70	1.69 ± 0.35	*t* = 0.385	0.705
Postoperative GFDI	2.04 ± 0.26^a^	1.99 ± 0.44^a^	*t* = 0.313	0.758
Preoperative AHD	6.39 ± 1.49	7.06 ± 2.07	*t* = −0.765	0.454
Postoperative AHD	5.83 ± 2.27^b^	6.92 ± 2.48^b^	*t* = −0.976	0.341

^a^ GFDI did not significantly increase postoperatively compared to preoperative levels in non-re-tear group (*P* = 0.405), but did show a significant difference in re-tear group (*P* = 0.042).

^b^ AHD before and after surgery did not show a significant difference in non-re-tear group (*P* = 0.530) and re-tear group (*P* = 0.823).

In partial repair group, correlation analysis was performed between postoperative Sugaya classification of rotator cuff healing and postoperative CMS as well as ULCA score. No statistically significant correlations were observed (*P* > 0.05). Further comparisons between partial repair group and debridement group revealed that, for both groups, preoperative GFDI showed no significant correlation with postoperative CMS or ULCA score (*P* > 0.05). However, preoperative AHD was significantly correlated with postoperative CMS and ULCA scores in both groups (*P* < 0.05) (Table 6).

**Table 6:** Correlation analysis between different factors in groups.

Group	Outcome measure	CMS	UCLA
Partial repair	Sugaya (I–V)	0.411^a^	0.028
		0.064	0.903
	Preoperative GFDI	0.307	0.249
		0.176	0.277
	Preoperative AHD	0.789*	0.806*
		<0.001	<0.001
Debridement	Preoperative GFDI	0.015	−0.036
		0.949	0.879
	Preoperative AHD	0.868*	0.872*
		<0.001	<0.001

a The number at the top of each unit is a correlation coefficient, and the value below is a p-value.

* Means significant difference (*P* < 0.05).

For all key outcomes with statistically significant results (*p* < 0.05), the estimated statistical power value ranged from 78.7% to 99% (with the minimum power value being 78.7%), closely aligning with the conventional threshold of 80%.

## 4 Discussion

### 4.1 Arthroscopic partial repair and debridement combined with acromioplasty

Arthroscopic partial repair and debridement combined with acromioplasty are both effective interventions for irreparable rotator cuff tears. Vogler et al. (13) demonstrated that debridement combined with acromioplasty significantly alleviate pain, enhance function, and increase patient satisfaction over a 10-year follow-up, although 26% of patients eventually required reverse shoulder acromioplasty. While debridement combined with acromioplasty alleviates pain-related strength loss and improves range of motion by removing inflammatory tissue and performing procedures like acromioplasty and greater tuberosity reverse-plasty, it does not restore shoulder coupling or biomechanics, making it unsuitable for patients with high expectations for strength and motion improvement (14). Burkhart suggested that partial repair can reduce tear size, enhance the biomechanical alignment of rotator cuff tear, and achieve a “functional tear” state by restoring the balance of anterior and posterior moments (15). Galasso et al. (16) reported that satisfaction with shoulder function and quality of life reached 90% in patients with partial repair after 7 years, despite the presence of a defect in superior shoulder joint coverage and a high risk of retear. Notably, studies indicate that repair failure does not significantly correlate with postoperative scores and functional improvement (17). Consistent with the re-tear subgroup comparison and Sugaya classification correlation findings in this study. The smaller size of retears compared to the original tears may explain the observed outcomes, as evidenced by a Korean study reporting a 34% reduction in tear size following partial repair (18) and another study noting a significant decrease in retears area (19). In this study, despite a retear rate of up to 66.7% in partial repair group, postoperative shoulder function scores, particularly for range of motion and strength, were significantly better than those in debridement group. Partial repair also helps maintain and slightly improve the acromiohumeral distance by restoring downward compression of the humeral head through torque restoration (3). Previous correlational studies have demonstrated that a larger preoperative acromiohumeral distance (AHD) significantly enhances glenohumeral head coverage after partial repair, thereby promoting postoperative functional improvement (20)—consistent with our findings regarding AHD in the current study. VAS scores were initially higher in partial repair group at two weeks postoperatively but decreased over time, showing no significant difference from debridement group after six weeks. This may be due to increased intraoperative tension from tendon release retraction sutures. The Global Fatty Degeneration Index (GFDI), a critical biomarker for evaluating postoperative outcomes (21), was exclusively preserved in the non-re-tear subgroup of partial repair group, whereas it demonstrated significant elevation across all other groups. These findings underscore the pivotal role of tendon healing in long-term functional recovery and highlight its necessity as a key consideration in the design of partial repair strategies. In both groups, partial repair aimed to restore shoulder joint function, while debridement combined with acromioplasty served as a palliative measure for symptom relief.

### 4.2 Characteristics of elderly patients with irreparable rotator cuff tears

Elderly patients with irreparable rotator cuff tears often experience localized reductions in shoulder bone mineral density and tendon degeneration. Chronic acromion impingement and reduced tendon blood supply are considered primary causes of this degeneration (22). Typically, there is no significant trauma at disease onset, or only a minor trauma history, with most patients unable to pinpoint the initial onset (23). Over time, injuries are compounded by factors such as tendon retraction, adhesion, and fatty infiltration. Tendon degeneration and decreased bone density hinder both intraoperative repair and postoperative tendon-bone healing. Additionally, the lack of tendon attachment stimulation leads to further bone density reduction in the footprint area (24), complicating repair efforts. Comorbidities like high cholesterol, elevated low-density lipoprotein, and hypertension, common in elderly patients, further increase the risk of repair failure (25). Recent studies indicate that severe rotator cuff injuries in elderly patients enhance the compensatory activation of intact muscles, such as the teres minor and deltoid, providing a foundation for postoperative functional improvement (26). Consequently, it is crucial to protect these intact muscles and stabilizing structures, like the rostral shoulder ligament, during surgery to prevent iatrogenic injuries from excessive loosening or molding. Inflammatory tissue debridement and acromioplasty can effectively alleviate pain, while preserving the superficial cancellous bone during the cleaning of the bony residue in the footprint area is essential to maintain a sufficient bone bed for anchor nail implantation (27). Postoperative administration of oral calcium and vitamin D can mitigate local bone mineral density loss and promote tendon-bone healing. Recently, bridging and balloon techniques have been developed, showing promise in enhancing postoperative shoulder function *in vitro* and in preliminary clinical trials, though some clinical findings diverge from basic research outcomes, warranting further investigation (28, 29). Arthroscopic partial repair and debridement combined with acromioplasty align better with the functional and lifestyle needs of most elderly individuals, offering advantages in operative time, cost, and rehabilitation duration. Notably, partial repair surgery significantly enhances shoulder function. However, it is unsuitable for elderly patients with high expectations for postoperative shoulder functional recovery.

There are several limitations in this study. First, the sample size was relatively small, which may introduce potential bias in outcome evaluation. Second, the mean follow-up duration was 14.1 months, which is relatively short and may not adequately capture long-term complications such as progression to arthropathy—particularly critical for rotator cuff pathology, where extended evaluation of functional durability remains necessary. Additionally, there was insufficient exploration of the long-term effects on shoulder function and quality of life prognosis, limiting a comprehensive understanding of the interventions' sustained impacts.

## 5 Conclusion

Arthroscopic partial repair yielded superior functional outcomes compared to debridement combined with acromioplasty in elderly patients with irreparable rotator cuff tears, particularly enhancing shoulder strength and range of motion while preserving AHD. Early postoperative pain should be anticipated. Preoperative AHD emerged as a predictor of functional recovery.

## Data Availability

The datasets presented in this study can be found in online repositories. The names of the repository/repositories and accession number(s) can be found in the article/Supplementary Material.
